# Neuroprotective effects of HTR1A antagonist WAY-100635 on scopolamine-induced delirium in rats and underlying molecular mechanisms

**DOI:** 10.1186/s12868-016-0300-9

**Published:** 2016-10-19

**Authors:** Yimin Qiu, Dongmei Chen, Xiaojing Huang, Lina Huang, Liang Tang, Jihong Jiang, Lianhua Chen, Shitong Li

**Affiliations:** 1 Department of Anesthesiology, Shanghai General Hospital, Shanghai Jiaotong University, 800 Dongchuan Rd., Minhang District, Shanghai, 200080 China; 2 Department of Pain Management, Shanghai General Hospital, Shanghai Jiaotong University, Shanghai, China; 3 Department of Anesthesiology and Pain Management, Shanghai General Hospital, Shanghai Jiaotong University, Shanghai, China

**Keywords:** Neurotransmitter, Neuroinflammation, HTR1A antagonist, Hippocampus, Amygdala, Delirium

## Abstract

**Background:**

Limited surveys have assessed the performance of 5-hydroxytreptamine receptor 1A and its antagonist WAY-100635 in pharmacological manipulations targeting delirium therapies. The purpose of this paper was to assess the central pharmacological activity of WAY-100635 in a rat model of scopolamine-induced delirium and its underlying mechanism.

**Results:**

A delirium rat model was established by intraperitoneal injection of scopolamine and behavioral changes evaluated through open field and elevated plus maze experiments. Concentrations of monoamines in the hippocampus and amygdalae were detected by high performance liquid chromatography. The effect of WAY-100635 on the recovery of rats from delirium was assessed by stereotactic injection of WAY-100635 and its mechanism of action determined by measuring mRNA and protein expression via real time PCR and western blotting methods. The total distance and the number of crossing and rearing in the elevated plus maze test and the time spent in the light compartment in the dark/light test of scopolamine-treated rats were significantly increased while the percentage of time spent in the open arms was decreased, showing the validity of the established delirium rat model. The measurement of the concentrations of noradrenaline, 3,4-dihydroxyphenylacetic acid, the homovanillic acid, 5-hydroxy-3-indoleacetic acid and serotonin concentrations in the cerebrospinal fluid (CSF) of scopolamine-induced delirium rats were significantly increased. The intra-hippocampus and intra-BLA injections of WAY-100635 improved the delirium-like behavior of rats by significantly reducing the expression of NLRP3 inflammasome and the release of IL1-β and IL8 into CSF.

**Conclusions:**

Taken together, these findings indicate that WAY-100635 may exert a therapeutic effect on post-operative delirium by controlling neurotransmission as well as suppressing neuroinflammation in the central nervous system.

## Background

Delirium is a multi-factorial transient neuro-psychiatric syndrome caused by well-defined predisposing and precipitating factors leading to severe outcome in hospitalized older patients [1]. Postoperative delirium (POD) is a common acute postoperative brain dysfunction that is associated with short-term complications such as an increase in mortality, morbidity, costs of medical care, poor functional recovery, postoperative cognitive dysfunction, deterioration in quality of life and length of stay, and long-term sequelae including persistent cognitive deficits and loss of independence [2, 3]. The incidence of POD is variable depending on the type of surgical and anesthetic procedures [4]. For post-surgical elderly patients, the incidence of POD is up to 80 % [5–7] due to frequent cognitive impairments following general anesthesia and surgery. Although POD is ominously associated with poor outcomes in patients, partly or completely reversing the behavioral changes is still possible if we understand the nature of the causative factors and the associated physiopathology [8].

The physiopathology of delirium at molecular level is unclear. Nevertheless, the current knowledge suggests that final common outcomes observed in POD are often associated with an alteration in neurotransmitter system that induces complex behavioral and cognitive changes [9]. Particularly, delirium results from dysfunctions of multiple systems of cholinergic neurotransmitters, which are characterized by significantly higher total serum anticholinergic activity (SAA) in older and young POD patients [10]. Central cholinergic pathways are involved in the control of cognitive processes which are responsible for the acquisition and retention of information as well as task performances. The dysfunctions of these pathways, induced by pharmacological administration of anticholinergic agents such as atropine and scopolamine or excitotoxic lesions, can produce a variety of behavioral effects and cause delirium [6, 11]. In general, the most commonly described neurotransmitter changes associated with POD include excess release of dopamine, norepinephrine and/or glutamate, as well as variable alterations of serotonin (5-hydroxytryptamine, 5-HT), histamine, and/or γ-aminobutyric acid (GABA) [12, 13]. Notably, neurotransmitters such as acetylcholine and serotonin have been found to play remarkably important functions in POD [14]. Nonetheless, the mechanisms by which 5-HT (serotonin) contributes to specific symptomatology of delirium remain an intense topic of investigation. Emerging evidence points to a role for the 5-hydroxytreptamine 1A (5-HT1A) receptor, which is highly expressed in limbic areas related to memory storage such as hippocampus, in the expression of specific symptoms of delirium. As a result, much attention has been paid to the HTR1A because its activation in discrete brain regions appears to be a sufficient stimulus to elicit specific symptoms of delirium.

Currently, pharmacological intervention has been well documented to reduce the incidences and severity of POD [15], among which antipsychotics are of better choice [5, 8]. However, the data to support specific pharmacological interventions for its treatment is still limited and current preventative strategies mainly consist of aggressive management of known risk factors [16]. Interestingly, the highly selective HTR1A antagonist, WAY-100635 (*N*-[2-(4-(2methoxyphenyl)-1-piperazinyl) ethyl]-*N*-(2-pyridinyl) cyclohexane carboxamide), has shown ability to block all of the effects induced by stimulation of this 5-HT receptor subtype [17, 18]. Therefore, studying the pharmacological effect of WAY-100635 in delirium could be valuable for therapeutic purposes.

Furthermore, a certain number of studies provided novel evidence that CNS (central nervous system) inflammation involving levels of cytokines such as IL-1β, IL-6 and IL-8 and pro-inflammatory molecules such as interferon gamma (IFN-γ) and anti-inflammatory molecules including insulin-like growth factor 1 (IGF-1) and IL-1ra may be associated with pathogenesis of delirium. Previous investigations showed that some important brain regions, including frontal lobes, diencephalon, hippocampus, amygdala and cerebellum define executive function, memory, attention, and play an important role in pathogenesis of delirium [19]. Despite abundant literature indicating the presence of monoamine transmitters in most brain regions that contain serotonergic neurons, little is known about their possible relationship with delirium symptoms and neuro-inflammation systems. Therefore, the neuropathogenesis for delirium, especially POD, remains largely to be determined through the establishment of animal models using cholinergic antagonists such scopolamine [20–22]. In addition, fewer attempts have been made to determine the expression of memory-related molecules in different brain regions of scopolamine-treated animals. Unearthing molecular mechanisms associated with delirium could enable the development of therapies due to better understanding of signaling pathways in play.

The main objective of the current pilot studies was to evaluate the ability of scopolamine to induce a delirium-like state in rats, to investigate changes in monoamine levels in different brain regions (including hippocampus and amygdala), and to determine whether rat behavioral activities can be reversed by the selective HTR1A antagonist, WAY-100635.

## Methods

### Animals

Healthy 2-month-old male SD rats, weighing 140–160 g were provided by the Second Military Medical University Animal Center (Shanghai, China) and housed in groups of three under controlled environment [18–20 °C, relative humidity of 55 ± 10 %, 12 h light/dark cycle (7:00 A.M. to 7:00 P.M.)] with free access to food and water. The maintenance and handling of the rats were performed in line with the Guide of Shanghai General Hospital for the Care and Use of Laboratory Animals. The study was reviewed and approved by the review board of the Shanghai General Hospital Animal Care and Use Ethics Committee. Each experiment occurred between 14:00 and 17:00 in order to avoid interferences of central neurotransmitter release caused by circadian rhythm. All measures were taken to reduce pain and the number of rats used in the study.

### Establishment of scopolamine-induced delirium rat model

A rat model of delirium was established via induction by intraperitoneal injection of different doses of scopolamine into rats according to their body weight. SD rats (n = 90) were randomly divided into control group (n = 15), saline group (n = 15) and four scopolamine treatment groups (n = 15 for each group) according to the concentration of scopolamine injected. The scopolamine concentrations tested were 0 mg/kg (control and saline groups), 0.3, 0.9, 1.2 and 1.8 mg/kg. Rats in the saline group were intraperitoneally infused with 1 ml physiological saline solution as vehicle. Thereafter, behavioral tests were performed to confirm memory impairment in rats.

### Monitoring and analysis of behavior

The tests used to assess the behavior of the rats included the open field test,the elevated plus-maze test of anxiety and the light/dark test which were performed as described below.

#### Open field test

The open field experiment was done as described elsewhere [23] using a self-made open-field test box (100 cm × 100 cm × 50 cm) uniquely made of Plexiglas with the base surface divided into 25 squares of 20 cm × 20 cm. The test was performed under dim light (40 W) in order to decrease the anxiety of the rats. Prior to test, the rats were weighed and gently put into the open field test chamber to get familiarized with the environment for 30 min to 1 h. After that, rats were gently and individually placed into the center of the open-field test box and were permitted free movement for 1 h. The movement parameters of each normal, scopolamine or saline solution injected rat were monitored and analyzed via a video-tracking system (Shanghai Mobile Datum Information Technology Company, Shanghai, China) each 5 min. The test box was cleaned after the removal of each animal in order to avoid that the remaining information of previous animals affects the next test results.

#### Elevated plus-maze test of anxiety

The maze was performed as described elsewhere [24]. The plus-maze apparatus consisted of two open arms, 50 × 10 cm, and two enclosed arms, 50 × 10 × 40 cm. Two arms were enclosed on three sides by 9 cm high transparent perspex and the other two arms were open. The rats were placed in the center of the lit looking the open arm and were assessed in a 5-min session. The rats behavior was recorded (Shanghai Mobile Datum Information Technology Company, Shanghai, China) and scored as the time spent by the rats in the closed and open arms. An arm entry was defined as two paws having crossed the dividing line between an arm and the central area.

#### Light/dark test

Immediately after the open-field test, all animals were submitted to the light/dark test using a rectangular Plexiglas box (44 × 8.5 × 25 cm) divided into light and dark sections that were separated by a door. Each rat was individually placed in the middle of the light compartment for 10 s exploration, after which, the way to the dim compartment was opened. The time spent in the dark and light compartments, the number of transitions, and the latency to enter the dark compartment were measured during a 10 min session.

### Measurement of the concentrations of monoamine neurotransmitters

#### Anesthesia, surgery and collection of rat cervical cerebrospinal fluid samples

After behavioral test, rats were put under anesthesia by intraperitoneal injection of pentobarbital (35 mg/kg). After anesthesia, a rat was placed in the horizontal position on a test table. The most caudal interspinous space was identified as a tactile landmark at the L3/L4 lumbar segment. After alcohol disinfection and shaving, a midline skin incision, approximately 2 cm in length, was made to expose the interspinous space at L3/L4 lumbar segment. After careful removal of the L3/L4 interspinous ligament, a metal needle was used for perforating the dural sac. A PP catheter was inserted at the junction of neck and chest cone segment from the dural tear to subarachnoid cephalic. After discarding the first 20 μl cerebrospinal fluid of each rat, 50 μl of cerebrospinal fluid was collected for the blank control group. After intraperitoneal injection of scopolamine or saline solution, 50 μl of cerebrospinal fluid was collected at specific periods (5–10, 15–20, 25–30 and 55–60 min) in an ice bath under dark conditions. Thereafter, the specimens were collected by centrifugation and immediately stored at −80 °C.

#### Microdialysis and HPLC analysis of monoamine neurotransmitters

Sample pre-treatment and detailed detection method were as previously described [25]. The concentrations of monoamine neurotransmitters including Norepinephrine (NE), 3,4-dihydroxyphenylacetic acid (DOPAC), homovanillic acid (HVA), 5-hydroxyindolacetic acid (5-HIAA), 5-hydroxytryptamine (5-HT), acetylcholine (Ach) and dopamine (DA) were quantified by HPLC analysis which was performed on an ESA (Chelmsford, MA) CoulArray HPLC instrument equipped with CouloChem III dual channel electrochemical detector channels utilizing an ESA 5041 carbon electrode. EZChrom software installed on a 32-bit PC was used for signal acquisition and data analysis and processing. The Capcell-MG2-C18 reverse column (150X2 mm, 3 µm) and a C-18 guard column were used for elution at a flow rate of 0.3 ml/min using a mobile phase containing 50 mM citric acid, 100 mM disodium hydrogen phosphate, 50 uM/L EDTA-2Na and 60 mg/L OAS, 6 % methanol. The mobile phase was unrecyclable, freshly prepared and filtered using a 0.45 um membrane. Quantitative analysis was accomplished using calibration curves obtained with standards of NE, DA, HVA, DOPAC, 5-HT, Ach, dopamine and 5-HIAA purchased from Sigma-Aldrich (Milwaukee, WI). Standard curves were established on the day of the experiment to ensure accuracy. The applied voltage was set to 350 mV and the guard electrode voltage was 400 mV with intensity of 50 uA. The analytical column and the electrode temperature was set to 32 °C using a single pump or the gradient-like mode analysis.

### Pharmacological effect of WAY-100635 on rat delirium

#### Animal grouping

SD rats were divided into 5 groups: scopolamine delirium group (model, n = 20), saline group (n = 20), scopolamine delirium group + WAY-100635 (model treated with WAY-100635, n = 40, 20 rats for hippocampus studies and 20 for amygdala studies), saline + WAY-100635 group (saline rats treated with WAY-100635, n = 40, 20 rats for hippocampus studies and 20 for amygdala studies) and WAY-100635 group (normal rats treated uniquely with WAY-100635, n = 40, 20 rats for hippocampus studies and 20 for amygdala studies).

#### Surgical procedure

Before surgery, rats were anesthetized with 35 mg/kg pentobarbital anesthesia according to the rat weight. These animals were implanted bilaterally with stainless-steel guide cannulae (11 mm) aimed above the hippocampus or BLA. The coordinates were determined by the rat brain atlas: for hippocampus, anteroposterior (AP), −3.4 mm from bregma, mediolateral (ML), −1.7 mm from midline, dorsoventral (DV), −2.7 mm from skull surface; for BLA, AAP = 2.8 mm caudal to bregma, Lat = 4.6 mm lateral to midline, DV = 8.7 mm ventral from the skull surface. The cannulae were affixed to the skull with dental cement, while two anchoring surgical screws and insect pins were inserted into the cannulae to maintain patency. After the cement was completely desiccated and solidified, two stainless steel stylets were employed for occluding the guide cannulae in the recovery period. Animals were individually housed and allowed to recover for 5–7 days before testing.

#### Drug microinjections

Microinjections of WAY-100635 purchased from Sigma (St Louis, MO, USA) were made 15 min prior to behavioral testing. During microinjections, rats were gently restrained by hand. After the stylets were removed from the guide cannulae by lowering stainless steel injector cannulae (30-G needle) with a length of 2 mm longer than the guide cannulae, microinjections were separately performed into either ventral hippocampus or BLA according to the animal grouping. Preliminary studies of injection of different concentrations of WAY-100635 solution (0.02, 0.05, 0.1, 0.2 mg/kg) were carried out on a limited number of rats to find out the optimal concentration of WAY-100635 to be administered. On this basis, WAY-100635 solution (0.1 mg/kg) was administered slowly in a total volume of 2.0 μl into the hippocampus and BLA over 60 s. Injection needles were left in place for an additional 60 s, followed by reinsertion of the stylets into the guide cannulae. All microinjections were performed bilaterally.

#### Behavioral test

After microinjections of WAY-100635, the open field test, the elevated plus-maze test of anxiety and the light/dark test were performed as described above.

#### Preparation of tissue lysates

For gene and protein expression analysis, rats were sacrificed by decapitation 2 h after injection of WAY-100635. After decapitation the brains were removed and immediately frozen on dry ice before dissection. To prepare tissue lysates, the prefrontal cortex (comprising motor cortex (areas 1 and 2) and cingulated cortex), striatum (dorsal), amygdala and hippocampus (caudal) were dissected and homogenized in 5 volumes of ice-cold buffer containing 50 mm Tris–HCl (pH 7.7), 150 mm NaCl, 1 % Nonidet P-40, 2 mm EDTA, 0.25 % sodium deoxycholate (DOC), 2 mm EGTA, 0.5 mm phenylmethylsulfonyl fluoride, 10 µg/ml leupeptin and incubated on ice for 30 min. After centrifugation at 14,000*g* for 20 min of the homogenate, the supernatant (tissue lysate) was aliquoted and congealed at −80 °C.

#### Western blotting

Lysates of hippocampus and BLA were heated at 80 °C for 30 min in RIPA buffer, and then centrifuged for 5 min at 10,000*g*. 20 µg total proteins was electrophoresed in a 10 % SDS-PAGE gel, and then electrically transferred onto PVDF membranes using a semidry method. The membranes were blocked for 1 h with a solution of TBS [containing 5 % (w/v) fat-free milk, and 0.5 % Tween 20], followed by overnight incubation at 1:1000 dilution at 4 °C with the following primary antibodies: anti-5HT1A receptor antibody (ab121032), Anti-5HT1B receptor antibody (ab85937), anti-5HT7 receptor antibody [EPR6271] (ab128892), anti-5HT3B receptor antibody (ab39629), anti-NLRP3 antibody (ab98151), anti-AKT1 (phospho S473) antibody (ab66138), anti-PI3K p85 (phospho Y607) antibody (ab182651), phosphor-S6K antibody, anti-muscarinic acetylcholine receptor 1 antibody (ab77098), anti-nicotinic acetylcholine receptor alpha 1 antibody (ab28489), anti-dopamine receptor D1 antibody (ab20066) and anti-GAPDH antibodies (all antibodies were purchased from Santa Cruz). GAPDH was used as endogenous reference for proteins. Thereafter, membranes were washed four times with PBS, and incubated with horseradish (HRP)-conjugated peroxidase anti-rabbit IgG (dilution 1:5000) for 1 h at room temperature. Detection was performed using the ECL-plus enhanced chemiluminescence system (ECL Amersham Biosciences) after autoradiographic exposure to HyperfilmTM.ECL (Amersham Biosciences). The program ImageMaster I-D (Pharmacia, Sweden) was used for quantitative determination of signals by densitometry.

#### Real time-PCR

Quantitative real-time PCR was used for detecting the mRNA expression level of 5-HT1A at gene level. Trizol extraction kit (Invitrogen, Carlsbad, CA) was employed for extracting total RNA from lysates of the frontal lobes, diencephalon, hippocampus, amygdale, parietal cortex and cerebellum. Subsequently, 1.5 μg extracted total RNA was converted into cDNA using First-Strand Synthesis System for RT-PCR (Invitrogen). The qRT-PCR experiment was achieved with the QuantiFast SYBR Green PCR Kit (Qiagen, Germany) according to the manufacturer’s protocol using a reaction volume of 20 μl. The PCR cycling condition was set as follows: 95 °C for 5 min, 40 cycles of 95 °C for 15 s, 60 °C for 15 s and 72 °C for 20 s. The fluorescence intensity was determined by the Bio-Rad CFX96™ Real-Time System. The relative levels of mRNA for the above receptor genes and 5-HT gene were normalized to GAPDH which was used as an endogenous gene. The primers used are presented in Table 1. Each test was run in triplicate.

**Table 1:** Primers used for real time PCR experiments

Gene	Primers	
	Forward (5′⋯3′)	Reverse (3′⋯5′)
HTR3B	TCTCCTGACCTGCCCTATGT	TCTGCCGGATGTGGTAAGTG
CHRNA4	CCTGACACGGGCAGTAGAAG	ACCACCACGTCCCTAGATCA
HTR7	CGTGATCAGCATCGACAGGT	TCACATTCTGAGCCCATCCG
DRD1	GAGGCTCCATCTCCAAGGAC	ACTGTGTGTGACAGGTTGGA
Chrm1	TACCTCCCTGTCACGGTCAT	GTGACCTCTCTGAGCTGCTG
HTR1B	GTGTGGGTCTTCTCCATCTCG	GCTTCCACATAGATACGGCCA
HTR1A	GACCACGGCTACACCATCTAC	CTGTCCGTTCAGGCTCTTCTT
	ATGGATGTGCTCAGCCCTGGTCAGG	CCTGACCAGGGCTGAGCACATCCAT
GAPDH	TGATGGGTGTGAACCACGAG	ATCACGCCACAGCTTTCCAG

#### Determination of IL-1β, IL-8 and TNF-α levels in CSF

IL-1β, IL-8 and TNF-α were quantified by sandwich ELISA using an IL-1β/IL-1F2 (R&D Systems, IL-1β/IL-1F2 Quantikine ELISA Kit), IL-8 and TNF-α (BioSource, Camarillo, CA) ELISA kits according to manufacturers’ guidelines.

### Statistical analysis

Data are presented as mean ± SD. GraphPad Prism version 5 was used to generate the graphics and perform statistical analysis. Groups were compared using one-way ANOVA followed by Bonferroni post tests for inter-group comparisons. Statistical significance was assigned to p values less than 0.05.

## Results

### Behavioral changes in scopolamine-induced delirium model

**Fig. 1:**
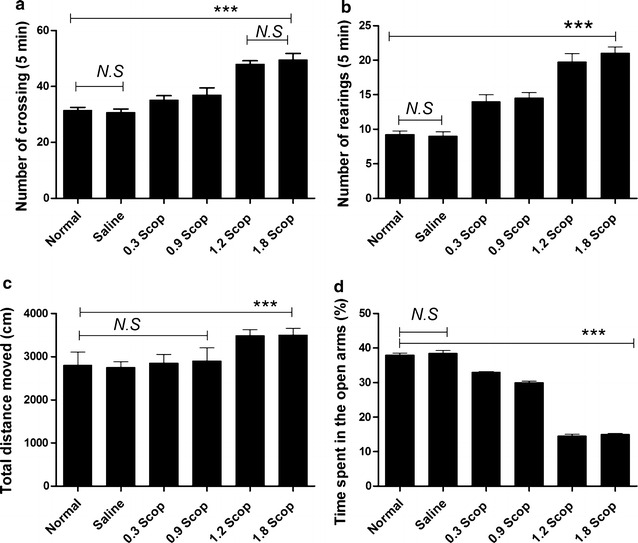
Scopolamine-induced delirium-like behavior of rats. **a** Number of crossing, **b** number of rearing and **c** total distance moved during the 5 min session in the open field test. **d** Percentage of time spent in the open arms during the 5 min session in the elevated plus maze test. Data are presented as mean ± SD. ***p < 0.001 by comparison against saline and control groups, *N.S* not significant

In open field test, locomotor activity was quantified in terms of total distance traveled after treatment with varied concentrations of scopolamine. Scopolamine treatment of 0.3 and 0.9 mg/kg did not elicit any differences in the total distance traveled relative to normal and control vehicle rats (Fig. 1a). Similarly, treatment with high scopolamine concentration led to significant increase of the number of crossing and the number of rearing compared to those of normal and saline-treated (1.0 ml/kg) rats. In elevated plus maze test, the percentage of time spent in the open arms of rats treated with 1.8 mg/kg was significantly decreased compared with saline-treated groups (F = 13.82, p < 0.001; Fig. 1d). As shown in Table 2, rats infused with 1.8 mg/kg scopolamine spent more time and ran long distances in the light compartment of the light/dark box compared to normal, saline and rats treated with low doses of scopolamine. Scopolamine treatment did not affect the number of transitions into the different compartments.

**Table 2:** Effects (mean ± SEM) of scopolamine rats in the light–dark transition model (n = 20 animals/group)

	Time spent (s)		Total distance moved (cm)		Number of transitions
	Light	Dark	Light	Dark	
Normal	140 ± 8.5	520 ± 7.45	1400 ± 304	2400 ± 235	9.7 ± 0.45
Saline	140 ± 7	528 ± 7.45	1350 ± 132	2340 ± 234	10.05 ± 0.75
Scopolamine treatment (mg/Kg)					
0.3	275.7 ± 10.	190 ± 8.5	1269 ± 200	2154 ± 159	9.2 ± 1.2
0.9	283 ± 3.5	182 ± 8.5	2000 ± 304	1564 ± 144	9.55 ± 0.5
1.2	350.9 ± 15	1790 ± 7	2150 ± 132	1812 ± 128	9.5 ± 1.5
1.8	409 ± 6.3	183 ± 10.32	2252 ± 150	1765 ± 235	9.01 ± 0.6

### Concentration of neurotransmitters in the cerebrospinal fluid (CSF) and brain regions of rats

**Fig. 2:**
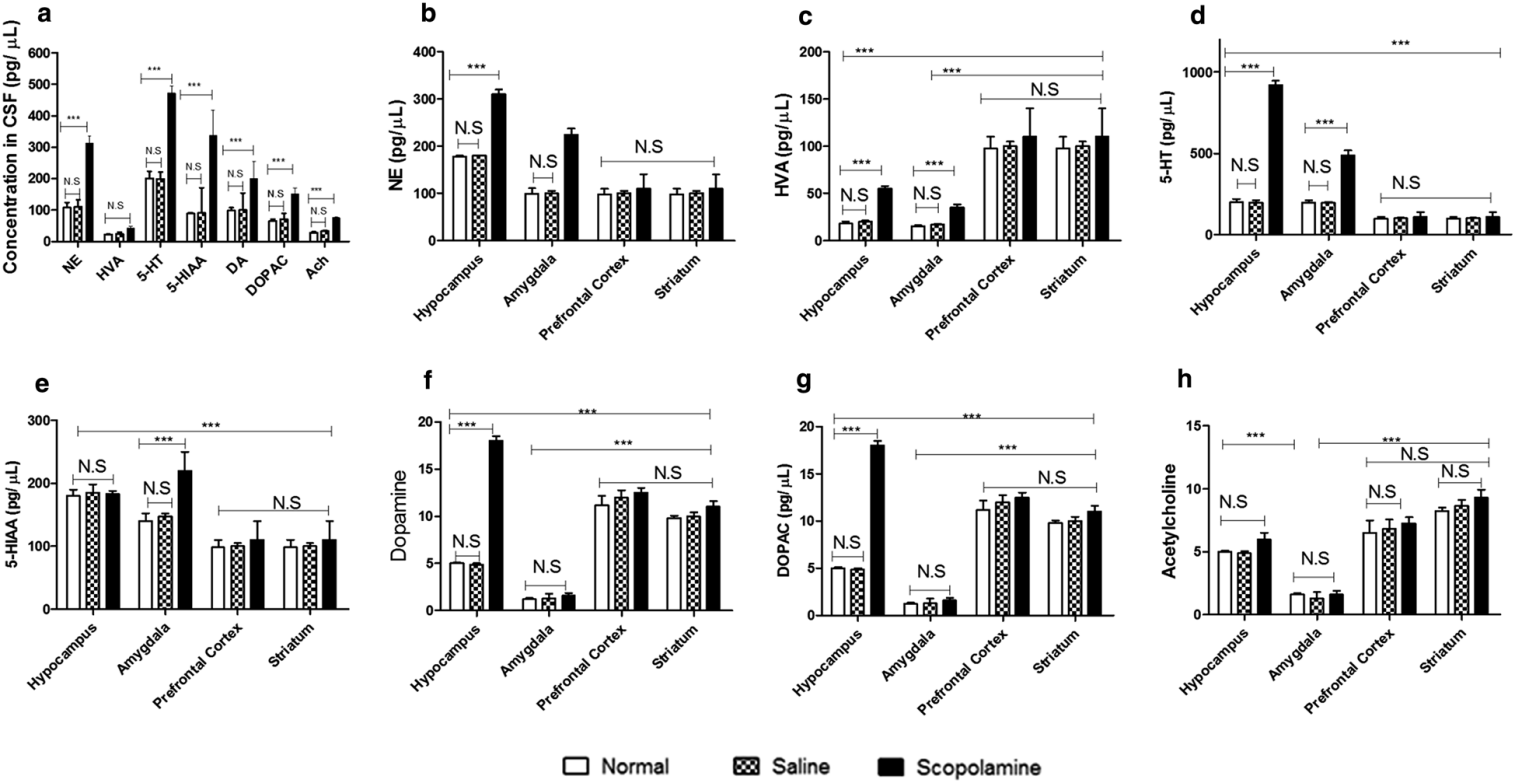
Effects of scopolamine on neurotransmitters in CSF and different delirium-associated brain regions. **a** The concentrations of neurotransmitters including NE, HVA, 5-HT, 5-HIAA, DA, DOPAC and Ach were determined in the CSF of normal, saline and scopolamine treated-rats. NE (**b**), HVA (**c**), 5-HT (**d**), 5-HIAA (**e**), DA (**f**), DOPAC (**g**) and Ach (**h**) were equally measured in hippocampus, amygdala, prefrontal cortex and striatum tissue homogenates by HPLC. Each value represents the mean ± SD of the parameters recorded, and the statistical analysis by Bonferroni’s test following one way ANOVA. ***p < 0.001 by comparison against indicated groups, *N.S* not significant

The concentrations of NE, Dopamine (DA), DOPAC, HVA, 5-HIAA, 5-HT and acetylcholine (Ach) in the CSF of scopolamine-induced impairment rats were 312.61 ± 22.24, 200.50 ± 53, 151.15 ± 18.93, 42.03 ± 5.98, 337.83 ± 80.31, 472.80 ± 52.92 and 75 ± 2 pg/μl, respectively, and were significantly increased compared to those of normal and saline-treated rats (Fig. 2a).

 Because it was reported that delirium is associated with brain regions that play an important role in executive functions (e.g., frontal lobes, diencephalon), memory (e.g., hippocampus, BLA), and attention (e.g., parietal cortex, cerebellum) [19], neurotransmitters including NE, DOPAC, HVA, 5-HIAA and 5-HT1A in hippocampus, amygdala, prefrontal cortex and striatum were measured. Compared to saline-treated rats, we found that scopolamine treatment significantly promoted the release of NE (p < 0.01; Fig. 2b), HVA (p < 0.01; Fig. 2c) and 5-HT (p < 0.001; Fig. 2d) in the hippocampus and BLA of high dose scopolamine-treated rats while no significant changes were recorded in the prefrontal cortex and the striatum. The concentration of 5-HIAA (p < 0.001; Fig. 2e) was increased in BLA but not in hippocampus while DA and DOPAC (p < 0.001; Fig. 2f) were increased in hippocampus but not in BLA. Meanwhile, the increased extent of 5-HT was higher than that of HVA, NE and DOPAC, especially in hippocampus of scopolamine-treated rats. The concentrations of DA, DOPAC, HVA and Ach were significantly higher in the prefrontal cortex and striatum compared to the amygdala while lower concentrations of NE, 5-HT and 5-HIAA were found in these regions. No significant differences were recorded between prefrontal cortex and striatum or between normal, saline or scopolamine treated rats in these regions.

### Activation of HTR1A in hippocampus and amygdala mediates the anxiogenic effect of scopolamine

**Fig. 3:**
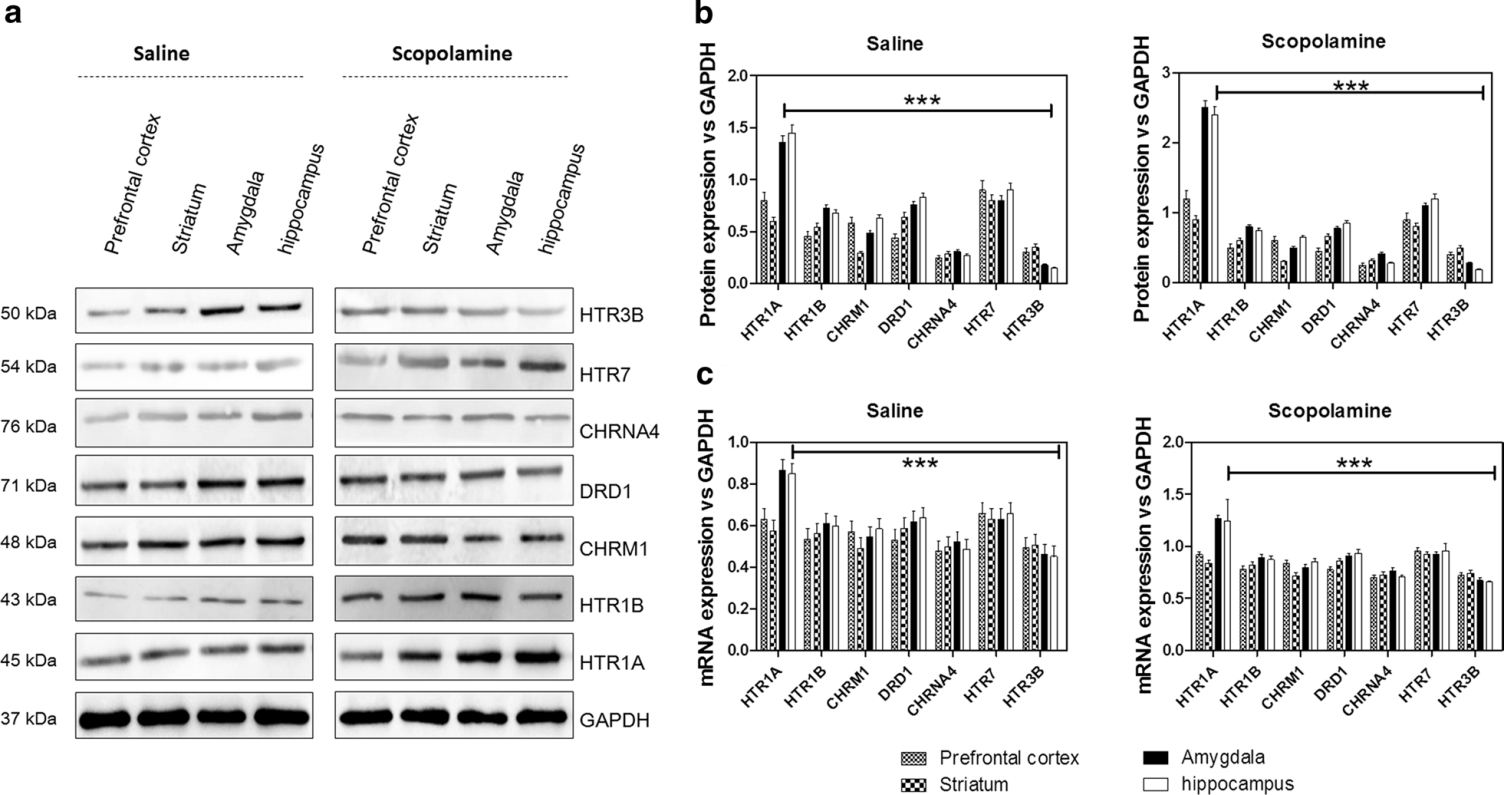
Expression of neurotransmitter receptors in different delirium-associated brain regions of control rats before and after scopolamine treatment. **a** Protein levels of neurotransmitter receptors were detected by western blot, **b** quantitative analysis of blots by densitometry, **c** mRNA level of neurotransmitters determined by qPCR; ***p < 0.001 by comparison against other neurotransmitter receptors

Based on scopolamine-induced increase of 5-HT in CSF, levels of 5-hydroxytryptamine receptor 3B (HTR3B), 5-hydroxytryptamine receptor 7 (HTR7), cholinergic receptor nicotinic alpha 4 subunit (CHRNA4), dopamine receptor D1 (DRD1), muscarinic acetylcholine receptor M1 (CHRM1), 5-hydroxytryptamine receptor 1B (HTR1B) and 5-hydroxytryptamine receptor 1A (HTR1A) were investigated in brain regions such as hippocampus, amygdala, prefrontal cortex and striatum. The result showed that for control rats with saline treatment or scopolamine-induced delirium rats, the protein level (Fig. 3a, b) and mRNA level (Fig. 3c) of HTR1A were significantly higher in hippocampus and BLA than other brain regions. Meanwhile, the expression of HTR1A in hippocampus and BLA of scopolamine-induced delirium rats was significantly increased when compared with those of saline-treated rats. The expression of HTR1B receptor was slightly increased in the four studied brain regions when compared with the saline treated rats, but no significant differences were recorded between groups or brain regions. Scopolamine treatment did not significantly affect the expression of HTR3B, HTR7, CHRNA4, DRD1 and CHRM1. These results indicate that the effect of scopolamine on memory impairment may be specifically mediated by high expression of HTR1A in hippocampus and amygdala.

### Effects of WAY-100635 on behavioral performance and biochemical phenotypes of scopolamine-induced memory impairment rats

**Fig. 4:**
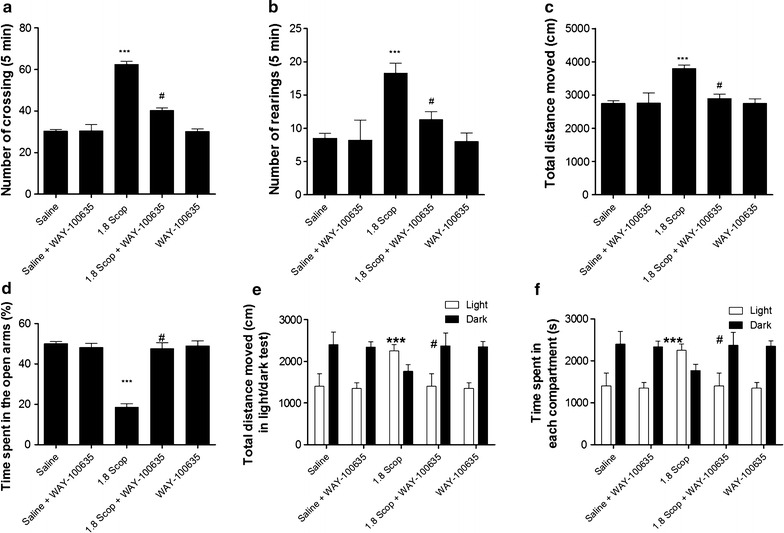
WAY-100635 reversed scopolamine-induced delirium-like behavior of rats. **a** Number of crossings, **b** number of rearing and **c** total distance moved during the 5 min session in the open field test, **d** Percentage of time spent in the open arms during the 5 min session in the elevated plus maze test, **e** total distance moved during the 10 min session in the light/dark test, **f** time spent in the open arms during the 10 min session in the light/dark test. Data are presented as mean ± SD. ***p < 0.001 by comparison against control rats and ^#^p < 0.001 by comparison against scopolamine-treated rats

**Fig. 5:**
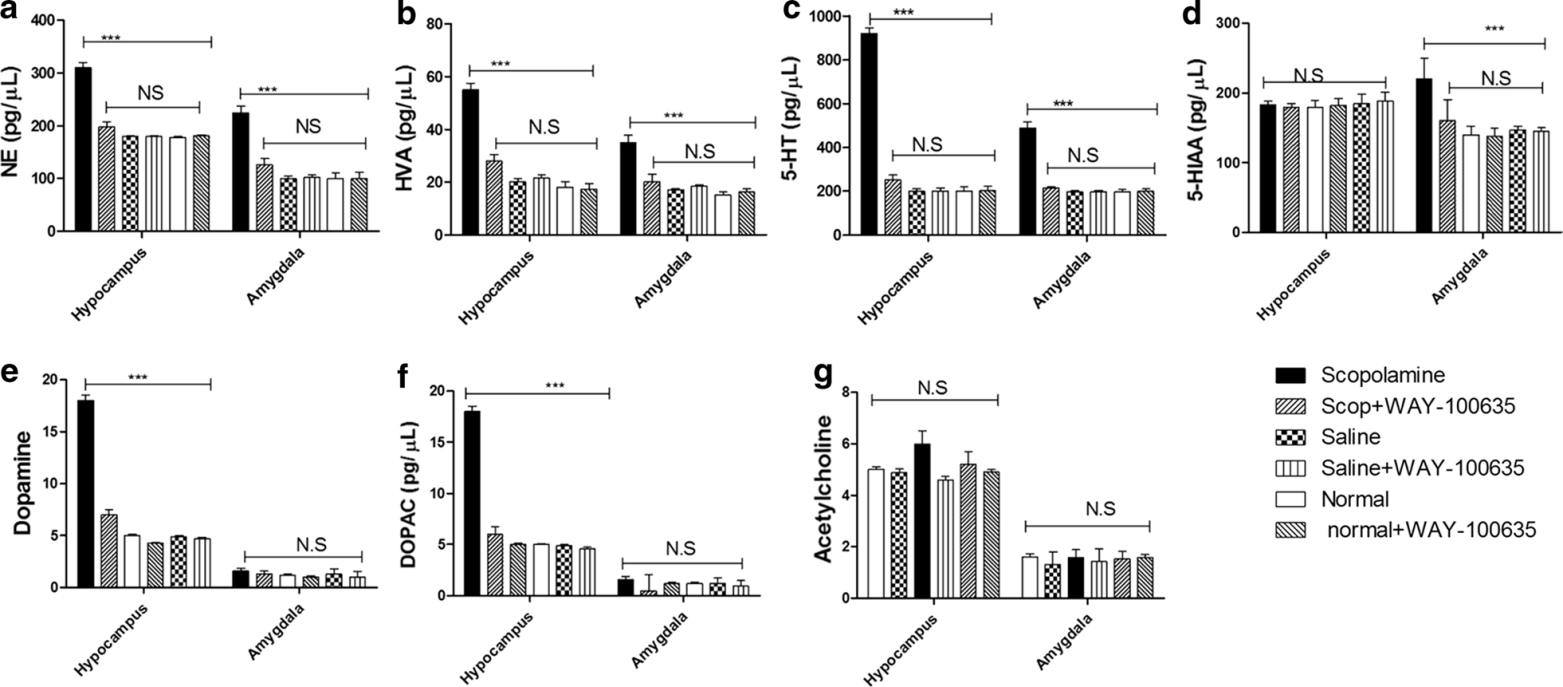
Effects of WAY-100635 on neurotransmitters in hippocampus and amygdala of delirium rats. The concentrations of neurotransmitters including NE (**a**), HVA (**b**), 5-HT (**c**), 5-HIAA (**d**), DA (**e**), DOPAC (**f**) and Ach (**g**) were equally measured in hippocampus, amygdala, prefrontal cortex and striatum tissue homogenates by HPLC. Each value represents the mean ± SD of the parameters recorded, and the statistical analysis by Bonferroni’s test following one way ANOVA. ***p < 0.001 by comparison against indicated groups, *N.S* not significant

Based on above results, stereotactic intra-hippocampus and intra–BLA injections of the HTR1A antagonist, WAY-100635, were performed in scopolamine-treated rats. Compared to scopolamine treatment, the microinjection of WAY-100635 (0.1 mg/Kg) to the delirium model group improved the number of crossing (p < 0.05; Fig. 4a), the number of rearing (p < 0.05; Fig. 4b) and the total distance (p < 0.05; Fig. 4c) in the open field test. In elevated plus maze test, the percentage of time spent in the open arms by the model group treated with WAY-100635 was significantly increased as compared with model groups (p < 0.05; Fig. 4d). Furthermore, we tested the effect of WAY-100635 in the light/dark test. Contrary to high dose scopolamine-treated rats, we recorded significant increase in total distance moved and in the number of entries in the dark compartment (Fig. 4e) and significant decrease in the time spent in the light compartment after WAY-100635 treatment were observed (p < 0.05; Fig. 4f). We equally tested the effect of WAY-100635 on the biochemical phenotypes and found that (Fig. 5) treatment of rats with WAY-100635 reversed the concentration of neurotransmitters in the hippocampus and amygdala of rats of delirium rats (1.8 mg/kg scopolamine).

### Effects of WAY-100635 on the expressions of cytokines and PI3K/Akt/mTOR pathway proteins

**Fig. 6:**
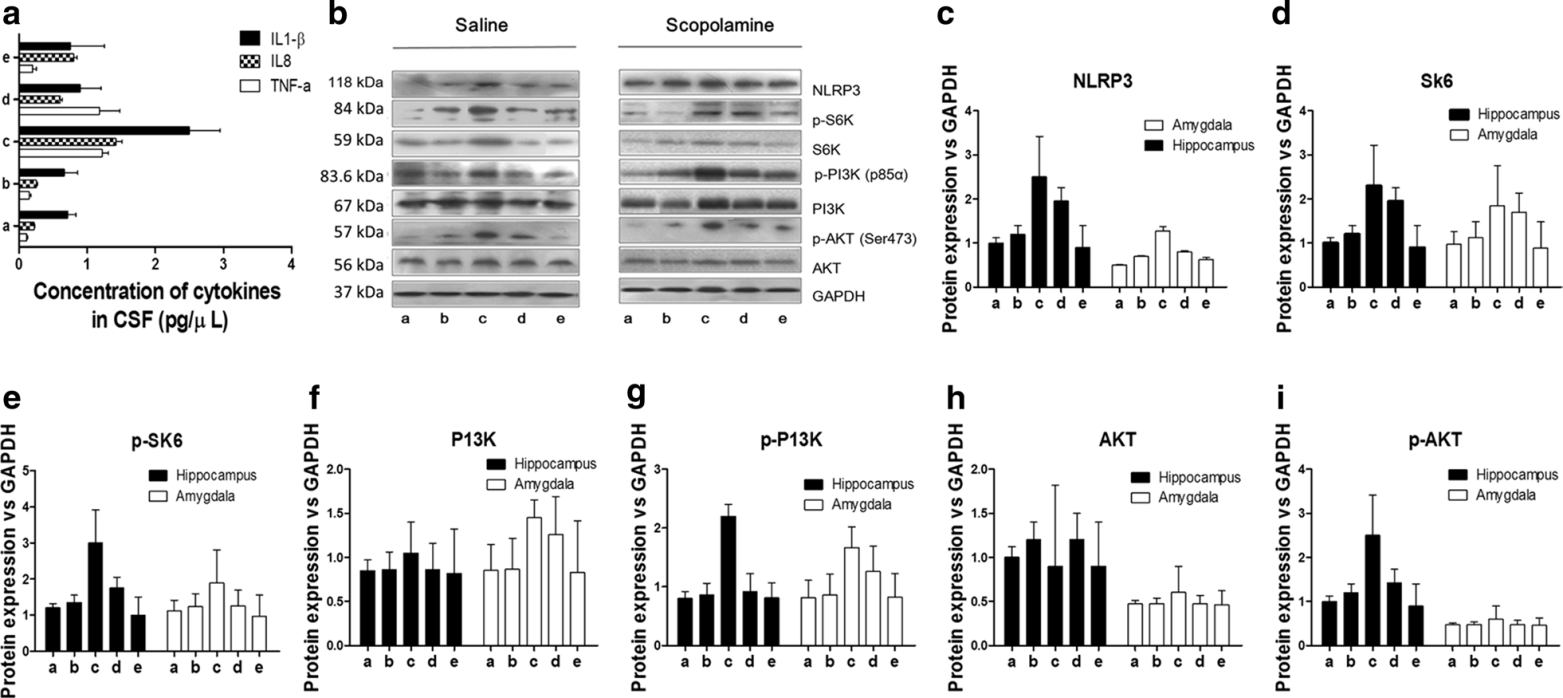
Effects of WAY-100635 treatment on cytokine release into CSF, NLRP3 expression and signaling pathways activation in hippocampus and BLA. **a** ELISA analysis of cytokine release into CSF of control, scopolamine-treated and WAY-100635 treatment groups. **b** Western blotting analysis of NLRP3 expression and phosphorylated PI3K [phosphor-PI3K (p85α)], AKT [phosphor-AKT (Ser473)] and S6K [phosphor-S6K] and GAPDH in hippocampus and BLA from different groups rats. **c**–**i** Quantitative values of western blot analysis. a: Saline, b: Saline + WAY-100635, c: scopolamine, **d** scopolamine + WAY-100635, e: normal + WAY-100635. ***p < 0.001 by comparison against other groups

Based on the association of neuroinflammation with delirium [26, 27], the levels of IL-1β, IL-8 and TNF-α in CSF were measured before and after WAY-100635 treatment. Intriguingly, it was found that the release of IL-1β and IL-8 into CSF was decreased after WAY-100635 treatment (Fig. 6a) while the levels of TNF-α were not significantly affected by WAY-100635 treatment. Accumulating data demonstrate that the release of IL-1β in CNS is induced by the activation of intercellular caspase-1, which is regulated by the NLRP3 inflamasome [28]. Therefore, the expression of NLRP3 in hippocampus and BLA tissue homogenates was measured after WAY-100635 treatment. The result (Fig. 6b, c) showed that, compared with that in hippocampus and BLA of scopolamine-induced delirium rats, expression of NLRP3 in such two brain regions was decreased after WAY-100635 treatment. Meanwhile, it was also found that phosphor-AKT (Ser473), phosphor-PI3K (p85α) and phosphor-S6K level were decreased after WAY-100635 treatment (Fig. 6c–i).

## Discussion

In humans, anticholinergic agents such as scopolamine are known to produce delirium-like states in patients and experimental animals [29–33]. For this reason, a scopolamine-induced delirium rat model was established in this report by intraperitoneal injection of 1.8 mg/kg of scopolamine in order to assess the delirium-related psychological behaviors. The results revealed the increase of crossing and rearing, and the decrease of the percentage of time spent in the open arms by scopolamine-treated rats comparatively to control rats treated with saline solution. In addition, the light/dark test revealed that after injection of 1.8 mg/Kg scopolamine, the distance moved and time spent in light compartment were increased while the transition between both light and dark compartment did not significantly changed. These observations potentiate the scopolamine-induced delirium rats as a useful delirium model for studying impairments of psychological behaviors and corroborated with the findings of K Nakamura, M Kurasawa and Y Tanaka [34] who showed that the intraperitoneal injection of 0.3 mg/kg of scopolamine engendered an impairment of task performance, increase in percent omission and decrease in percent correct, demonstrating behavioral discrepancies and a reduced arousal or vigilance.

Nowadays, it is believed that CSF analysis in search of the pathogenesis of delirium has great potential to advance understanding of POD pathophysiology due to its proximity to the brain and its immune-privileged position behind the blood brain barrier (BBB) [26]. In order to investigate the effect of scopolamine on the expression of neurotransmitters, we performed HPLC analysis of the CSF and found that NE, DOPAC, HVA, 5-HIAA and 5-HT concentrations in CSF of scopolamine-induced delirium rats were significantly increased, with a particular emphasis for 5-HT. For providing consistent data of these neurotranmitters on delirium, we further studied the release of some neurotransmitters in hippocampus and BLA. The release of NE, HVA and 5-HT in hippocampus and BLA of scopolamine-induced delirium rats increased considerably by comparison with the control group. 5-HIAA was increased in BLA but not in hippocampus while quite the opposite was recorded for DOPAC. In addition, the increased extent of 5-HT was higher than that of other neurotransmitters, especially in the hippocampus. These results are consistent with previous findings suggesting that hippocampus is selectively enmeshed in contextual memory consolidation while the amygdala is mostly engaged in modulating the consolidation of memory for emotionally arousing experiences [35]. The present results equally support an association between higher level of 5-HT in CSF and delirium, indicating that more definitive studies of the relationship between 5-HT level and delirium are now required [36]. In addition, in the two brain regions, increase of mRNA and protein levels of HTR1A was discovered in the present study. This novel finding indicates that serotonergic neurotransmissions located on hippocampus and amygdale function in controlling the pathogenesis of delirium [37], implying that antagonism at 5-HT receptors must be an important tool in treating delirium as it was previously demonstrated for 5-HT2 receptors [38]. To verify this hypothesis, we proceeded to microinjections of WAY-100635, a potent and selective HTR1A antagonist, into the hippocampus and BLA of scopolamine-treated rats. It was observed that, compared to control rats, treatment of WAY-100635 improved the delirium-like behavior of rats. As discussed above, the serotonergic system may vary depending on the severity of delirium symptoms, but HTR1A antagonist treatment affecting the function of hippocampus can influence the modulation of a variety of neurophysiological changes [38].

Furthermore, previous researchers indicated that cholinergic system and inflammation are common pathways involved in delirium pathophysiology and incriminated IL-1β as the major inflammatory factor [26, 27]. In order to investigate the possible connection of WAY-100635 with inflammatory pathways in the occurrence of delirium, a key pro-inflammatory cytokine IL-1β in CSF was analyzed before and after WAY-100635 treatment. It was observed that release of IL-1β into CSF was decreased after WAY-100635 treatment. In addition, the NLR family, pyrin domain-containing 3 (NLRP3) inflammasome, an important upstream regulator of IL-1β, and phosphor-AKT (Ser473), phosphor-PI3K (p85α) and phosphor-S6K expressions were decreased. These results implied that WAY-100635 improves the rat delirium systems by inhibiting the PI3K/AKT/mTOR activation in hippocampus and BLA to subsequently prevent NLRP3-mediated IL-1β release into CSF. Consistent with previous findings, this study approves that the PI3K/Akt signal pathway may be involved in both the non-spatial and spatial cognitive impairment and its reversal using WAY-100635 may be useful in alleviating delirium symptoms.

## Conclusions

Taken together, the present findings indicate that central nervous system serotonergic neuronal activity and neuro-inflammation in hippocampus and BLA could be involved in the pathogenesis of scopolamine-induced delirium model of rats, which may be important to understand the behavioral pathologies of delirium. Meanwhile, these data yield a novel insight that WAY-100635, the selective antagonist of HTR1A could be clinically useful in the ultimate treatment of POD.

## Abbreviations

POD: postoperative delirium
HPLC: high performance liquid chromatography
NE: noradrenaline
DA: dopamine
Ach: acetylcholine
DOPAC: 3,4-dihydroxyphenylacetic acid
HVA: homovanillic acid
5-HIAA: 5-hydroxy-3-indoleacetic acid
5-HT: serotonin
BLA: amygdala
PCR: polymerase chain reaction
CSF: cerebrospinal fluid
i.p.: intraperitoneally
IL-1β: interleukin-1 beta
NLRP3: NOD-like receptor family, pyrin domain containing 3
PI3K: phosphatidylinositol-3-kinase
Akt: protein kinase B (PKB)
mTORC1: mammalian target of rapamycin complex 1
